# Identifying the seeds of heterotic pools for Southern and Eastern Africa from global elite spring wheat germplasm

**DOI:** 10.3389/fpls.2024.1398715

**Published:** 2024-06-27

**Authors:** Carus John-Bejai, Richard Trethowan, Isobella Revell, Stephan de Groot, Lindani Shezi, Francois Koekemoer, Simon Diffey, Jacob Lage

**Affiliations:** ^1^ Wheat Breeding, KWS UK Ltd, Thriplow, United Kingdom; ^2^ The Plant Breeding Institute, School of Life and Environmental Sciences, The University of Sydney, Narrabri, NSW, Australia; ^3^ Wheat Breeding, Sensako (Syngenta), Bethlehem, South Africa; ^4^ Apex Biometry, Fremantle, WA, Australia

**Keywords:** heterotic, yield, CIMMYT, Africa, wheat

## Abstract

Hybrid breeding can increase the competitiveness of wheat (*Triticum aestivum* L.) in Sub-Saharan Africa by fostering more public-private partnerships and promoting investment by the private sector. The benefit of hybrid wheat cultivars in South Africa has previously been demonstrated but due to the high cost of hybrid seed production, hybrid breeding has not received significant attention in the past decade. Considering the renewed commitment of the private sector to establish wheat as a hybrid crop globally, coupled with significant research investment into enhancement of outcrossing of wheat, hybrid wheat breeding in Southern and Eastern Africa should be revisited. Our study aimed to identify genetically distinct germplasm groups in spring wheat that would be useful in the establishment of heterotic pools targeting this region. Multi-environment yield testing of a large panel of F1 test hybrids, generated using global elite germplasm, was carried out between 2019 and 2020 in Argentina, Africa, Europe, and Australia. We observed significant genotype by environment interactions within our testing network, confirming the distinctiveness of African trial sites. Relatively high additive genetic variance was observed highlighting the contribution of parental genotypes to the grain yield of test hybrids. We explored the genetic architecture of these parents and the genetic factors underlying the value of parents appear to be associated with their genetic subgroup, with positive marker effects distributed throughout the genome. In testcrosses, elite germplasm from the International Maize and Wheat Improvement Center (CIMMYT) appear to be complementary to the genetically distinct germplasm bred in South Africa. The feasibility of achieving genetic gain via heterotic pool establishment and divergence, and by extension the viability of hybrid cultivars in Sub-Saharan Africa, is supported by the results of our study.

## 1 Introduction

Wheat cultivation is not a recent phenomenon in Sub-Saharan Africa; we find evidence of Emmer wheat (*Triticum turgidum* subsp. *dicoccum*) cultivation in Ethiopia 5000 years ago (Melese et al., 2019) and reports of bread wheat (*Triticum aestivum* L.) exports from the Cape provinces of South Africa to India in 1684 (Van Niekerk, 2001). It is likely that the first wheat breeding conducted in the region occurred in South Africa (1891) (Smit et al., 2010). Investment into the genetic improvement of wheat in South Africa has been maintained to the present day, producing tangible returns; dryland wheat production increased from 0.5 tonnes per hectare in 1936 to 3.5 tonnes per hectare in 2015 (Nhemachena and Kirsten, 2017). The role of local private industry in achieving this cannot be understated; between 1891 and 2013, 171 wheat varieties were released by local companies compared to 72 by local public institutions (Nhemachena and Kirsten, 2017). Of the three main wheat breeding entities described by Smit et al. (2010), two trace their origins back to the establishment of local companies; 1) Sensako (now functioning as part of Syngenta) established in the 1960s and 2) Pannar (now functioning as part of Pioneer), who initiated wheat breeding programmes in the 1990s.

The decline in wheat prices after May 2022 in South Africa makes it unlikely that the area under wheat cultivation will expand further to compensate for lower yields in recent seasons (Esterhuizen and Caldwell, 2023). Coupled with this, the current economic climate imposes a constraint on wheat importation to mitigate reduced production (Esterhuizen and Caldwell, 2023). Yield instability and volatile market prices are not unique to South Africa; in Kenya, wheat is the second most important source of calories after maize, but on average only 300,000 metric tonnes are produced annually (Ly et al., 2022). Kenya is therefore dependent on imports of grain to meet domestic demand; 32 percent of the wheat consumed in Kenya in 2020 originated from Russia (Ly et al., 2022). Private sector investment in wheat breeding in Kenya is less than in South Africa and past genetic gains have been predominantly driven by a single public institution in collaboration with foreign public breeding programmes. Maintaining or improving rates of genetic gain in Kenya are constrained by rapid changes in fungal rust pathogens (Macharia and Ngina, 2017) and extreme climate variability (Ly et al., 2022).

Despite being the second largest producer of wheat in Africa, Ethiopia has imported substantial amounts to meet domestic demand over much of the past century (Senbeta and Worku, 2023). To reduce their dependency on scarce foreign currency reserves, the government of the country implemented an irrigated wheat initiative in 2019 which produced tangible results as early as 2022 when Ethiopia achieved wheat self-sufficiency (Effa et al., 2023). In 2023 even higher production was realized which enabled the country to become a net exporter of wheat (Effa et al., 2023). An efficient water management system will be crucial in ensuring the sustainability of the current initiative, a challenge considering current and predicted climatic conditions (Effa et al., 2023; Senbeta and Worku, 2023). Wider deployment of new improved varieties can reduce this vulnerability; sustained introductions of CIMMYT and The International Center for Agricultural Research in the Dry Areas (ICARDA) material over the past 50 years have continued to redefine the yield potential and stability of Ethiopia’s wheat production systems (Tadesse et al., 2022).

While public breeding institutions have made a large contribution to yield progress in Sub-Saharan Africa, Tadesse et al. (2018) stressed that low levels of public-private cooperation in wheat production and breeding must be addressed if sustainable production is to be achieved and maintained in Sub-Saharan Africa. Multinational seed company engagement has remained low for wheat compared to maize (*Zea mays*) in South Africa, because maize has the advantage of being a hybrid crop and consequently is more attractive for investment (Koekemoer et al., 2011). Koekemoer et al. (2011) suggested that hybrid wheat could be a viable approach to ensuring that wheat production remains competitive and attracts the investment in research and development required to meet current and future challenges.

Prior to our study, commercial heterosis for grain yield (i.e. performance relevant to the best inbred commercial check) as high as 22 percent was observed in South Africa by breeders at Sensako (Koekemoer et al., 2011). Despite the increased cost associated with the production of F1 seed, we anticipate that this level of yield improvement can make wheat more attractive to South African farmers, thus compensating for the reduced area under wheat cultivation. Further, we expect that once genetically distinct pools of inbred parental lines are established, hybrid wheat breeders will be able to respond quickly to changes in abiotic and biotic conditions an attractive prospect for South Africa, Ethiopia and Kenya.

We anticipate that South Africa can play a significant role in the effective deployment of hybrid wheat cultivars in Sub-Saharan Africa for two reasons; 1) there is already infrastructure and local expertise in hybrid wheat breeding available and 2) wheat is grown across a wide range of agro-ecologies in South Africa (irrigated and rainfed; winter rain or summer rain) which should enable the development of hybrids with adaptation to specific environments and/or broad adaptation across regions. The crux of hybrid wheat in South Africa has been the high cost of F1 seed production; since 2010, hybrid wheat breeding at Sensako has been a minor component of their crop portfolio (Koekemoer et al., 2011).

Recent renewed interest in hybrid wheat breeding by the private sector globally has led to extensive research. As a consequence, our understanding of the genetic factors influencing outcrossing potential in wheat and the implications for large scale F1 seed production has improved significantly (Boeven et al., 2018; Schneider et al., 2024). Coupled with this, a wide range of male sterility systems have been developed with commercial seed production in mind (Whitford et al., 2013; Sun et al., 2019). The wheat breeding community is therefore well placed to revisit heterosis for grain yield in Sub-Saharan Africa.

Mackay et al. (2021) emphasized that the search for new sources of useful genetic variation for hybrid breeding must focus on the heritable component of trait variation, additive genetic variance. The additive effect of a parental line on a given trait is typically described as its general combining ability (GCA) and many authors emphasize that this component is solely attributable to additive effects; conversely specific combining ability (SCA) captures the non-additive effects of dominance and epistasis. However, GCA values are derived from the average testcross effect of alleles, which are a function of the genotypic values of homozygotes as well as heterozygotes and therefore captures both additive and dominance variance (Bernardo, 2002). Testcross effects are dependent on the testers used and the extent to which dominance is captured in GCA (i.e. the extent to which dominance acts in an additive manner) is influenced by the relationship of the test germplasm to the corresponding testers. Breeding for heterotic pools diverges germplasm pools so that the ratio of GCA to SCA increases over time due to the conversion of non-additive effects, initially associated with SCA, into GCA effects (Labroo et al., 2021). This makes the performance of parents in test hybrids more predictable/heritable (Schulthess et al., 2017). This divergence can be achieved through cycles of reciprocal recurrent selection irrespective of which lines are initially allocated to each heterotic pool; the use of genetically distinct founders can however, accelerate this process considerably (Labroo et al., 2021). Herein lies the focus of the investigations in the present study.

Using a diverse set of elite spring wheat germplasm as male and female parents, we attempted to ascertain the extent of additive genetic variance for grain yield in Sub-Saharan Africa in a defined set of germplasm by large scale testing of their test hybrids. This approach was taken with the aim of identifying distinct genetic subgroups within the set of the inbred parents and assessing the extent to which these groups can contribute positive additive genetic effects for grain yield to test hybrids. In addition, we hoped to ascertain the similarity between environments in South America, Europe, Australia, East Africa, and South Africa to provide insight into the extent to which breeding efforts in South America, Europe and Australia are transferable to Sub-Saharan Africa. We aimed to provide regional breeders with 1) descriptions of relevant genotype by environment interactions (GxE), 2) the distribution of additive allelic (testcross) effects for grain yield across the wheat genome and 3) a characterization of existing germplasm for its utility in heterotic pool formation targeting the region. The extent to which we were able to achieve these objectives is discussed.

## 2 Methods

### 2.1 Plant material and experimental design

For consistency, individual trial locations in a year will be referred to as trial sites in the remainder of this paper. The experimental material consisted of 722 F1 test hybrids of spring wheat that were produced by KWS UK Ltd and the University of Sydney using 131 elite cultivars from across the globe as pollen parents and 25 nuclear male sterile female parents developed by either KWS UK Ltd. or University of Sydney (**Supplementary Table 1**). All parents were assumed to be fully inbred.

**Figure 1:**
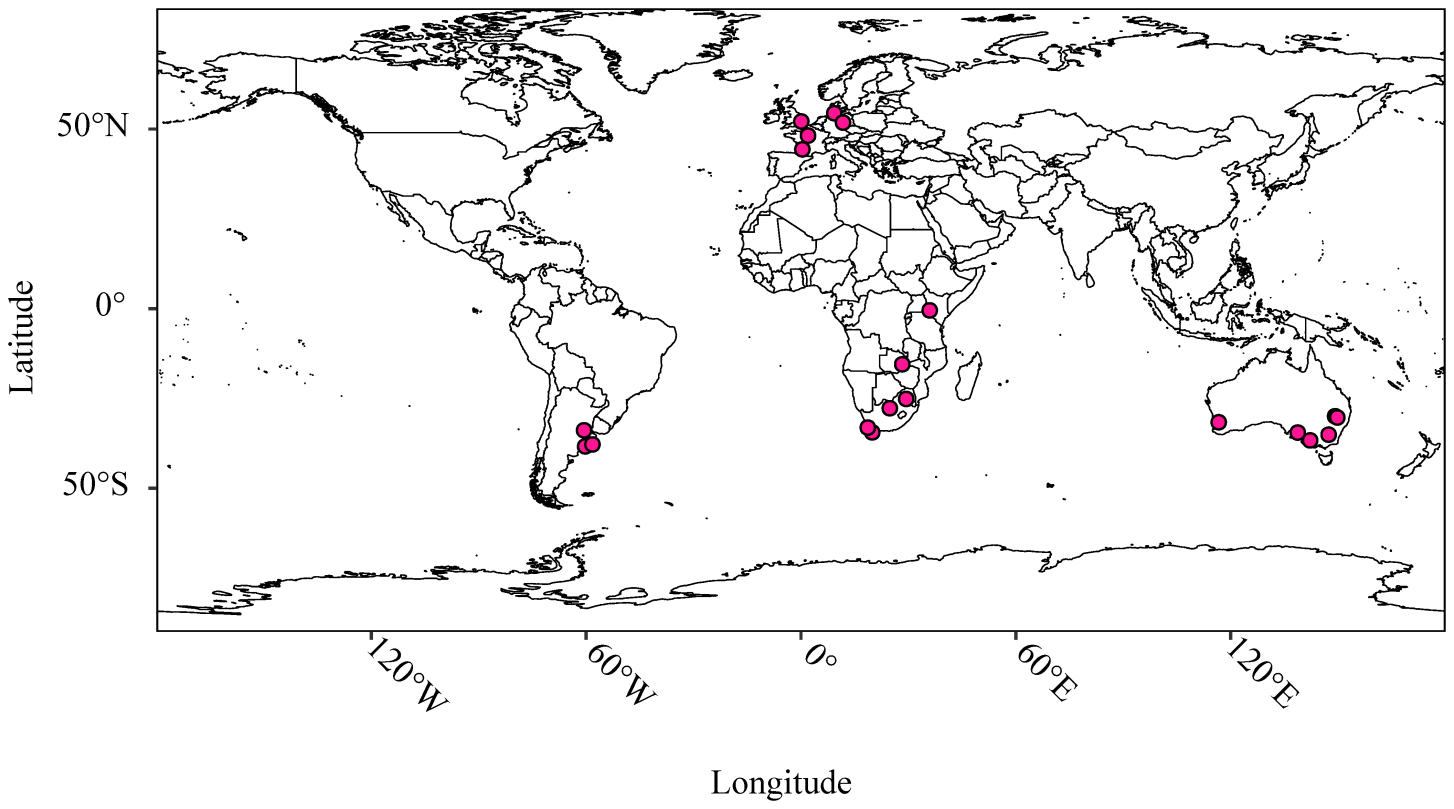
Location of trial sites (filled circles) used in the present study.

**Figure 1** provides a graphical representation of the location of trial sites; further details are provided in **Supplementary Table 8**. Materials were arranged in partially replicated (p-rep) designs; a subset of test hybrids allocated to each trial site in a sparse multi-environment trial (MET) design (Cullis et al., 2020) while attempting to optimize the concurrence of male and female parents across the MET (**Supplementary Tables 2–4**). Five elite inbred cultivars (representing materials from the International Maize and Wheat Improvement Center (CIMMYT), South America and South Africa)were included at all 24 trial sites as checks (**Supplementary Table 2**). One or more local cultivars relevant to each testing site were also included in the panel of checks. Yield trials were conducted at each test site following the standard agronomic procedures used in each region including application of fertilizers and agrochemicals. Meteorological data accessible by KWS via a service provider DTN Clear Ag® were obtained for each trial site from the date of sowing to the date of harvest; variables for which data was obtained are described in **Supplementary Table 9**.

### 2.2 Genetic characterization of parents

Genotypic data was generated for the panel of parents by single nucleotide polymorphism (SNP) analysis on the Axiom 35K Breeder’s Array (Allen et al., 2017). Additionally, the panel was genotyped using KWS’ proprietary Kompetitive Allele Specific PCR (KASP) markers for loci with an established effect on adaptation and disease in KWS’ germplasm; the target locus (using the nomenclature proposed by Boden et al. (2023)) for each of these markers and a description of their associated phenotypic effect is provided in **Supplementary Table 6**. Markers with >50% heterozygous calls and/or >10% missing data were excluded from analyses, giving a total of 7758 genome wide SNPs. Physical map positions for available SNPs, based on the wheat reference genome assembly of cv. ‘Chinese Spring’ (IWGSC et al., 2018, RefSeq v1.0), were downloaded from ‘CerealsDB’ (Wilkinson et al., 2020) and used for the visualization of additive marker effects.

A Rogers’ genetic distance matrix (Reif et al., 2005) was generated for the panel and principal coordinate analysis used to derive the first two principal coordinates that differentiated the germplasm using KWS’ proprietary code within the R computing environment (R Coreteam, 2021). To further characterize population structure within the panel, 3-fold cross validation was employed within the software tool ADMIXTURE (Alexander et al., 2009) to first determine the optimal number of ancestral populations to fit; the minimum and maximum number of populations was set to two (2) and ten (10) respectively and was guided by the number of breeding programmes and geographical regions from which the parents originated. The number of populations within this range that minimized the estimated prediction error (**Supplementary Table 11**) was taken as the optimal number (Alexander et al., 2009); genotypes were then assigned to the inferred number of populations within the ADMIXTURE tool. For principal coordinate and ADMIXTURE analyses, a single marker in each pair showing complete linkage (r^2^ = 1) were excluded from the genotype dataset.

### 2.3 Individual trial analysis

Linear mixed models were used to fit a statistical model to yield data collected from each trial site using the package ASReml (Butler et al., 2009) within the R computing environment (R Coreteam, 2021). Models for each trial site were developed in three stages. The first stage was the creation of a base model where trial design factors (row, column, and block) were fitted as random effects, hybrid line effects were fitted as random effects and non-hybrid line (checks) effects were fitted as fixed effects. The base model was expanded in the second stage by considering terms which captured field spatial variability using the process described by Gilmour et al. (1997) and the diagnostics of Stefanova et al. (2009). The final stage entailed incorporating pedigree information for the hybrid lines in the form of the numerator relationship matrix. This involved partitioning hybrid line effects into additive and non-additive effects with the numerator relationship associated with the former (Oakey et al., 2006). We use the terms accuracy and reliability as defined by Mrode and Thompson (2005) in the present study; reliability is taken as synonymous with narrow sense heritability (*h^2^*).

### 2.4 Multi-environment trial (MET) analysis and GBLUP for grain yield

The final models developed for the 24 trial sites were advanced to a MET analysis using factor analytic mixed models (Oakey et al., 2007; Smith and Cullis, 2018). A factor analytic mixed model of order 4 (FA4 model) was used to estimate the additive genetic variances and covariances between trial sites. The matrix of estimated genetic variances and covariances between trial sites was converted to an additive genetic correlation matrix and hierarchical clustering was performed to group trial sites into clusters. The MET model was extended by replacing the numerator relationship matrix with a genomic relationship matrix (Tolhurst et al., 2019). Using this model, for grain yield we obtained best linear unbiased predictions (BLUPs) for parental and hybrid additive genetic effects. Additionally, marker effects for grain yield were estimated from this model.

For specific case studies, the stability of the additive genetic effect of parents across environmental clusters was assessed by calculation of Shukla’s stability variance (Shukla, 1972) using the package statgenGxE (Van Rossum, 2024) within the R computing environment (R Coreteam, 2021).

### 2.5 Usefulness simulations

Utilizing marker effects for grain yield in a given trial site, the genotypic data of the parents and a consensus genetic map (Wang et al., 2014), the genotypic profile of 250 doubled haploid individuals were simulated for specific cross combinations. Genomic estimated breeding values (GEBVs) were estimated for individuals by multiplying the marker matrix by the estimated marker effects and by then adding the mean grain yield of the respective trial. Applying a selection intensity of 10%, the usefulness criterion (UC) (Schnell and Utz, 1975) for these populations was calculated using the following model.


UC=μ+i h σ

Where μ is the genetic mean (mean of the simulated offspring), σ is the genetic standard deviation of the progeny, i is the selection intensity applied and h the selection accuracy [assumed to be one (1) as selection is based on marker effects (Zhong and Jannink, 2007)]. Simulations were carried out using KWS proprietary code within the R computing environment (R coreteam, 2021) and analysis was restricted to African trial sites; all possible interpopulation and intrapopulation single cross populations involving South African germplasm were simulated.

## 3 Results

### 3.1 Genetic variance for grain yield

**Figure 2:**
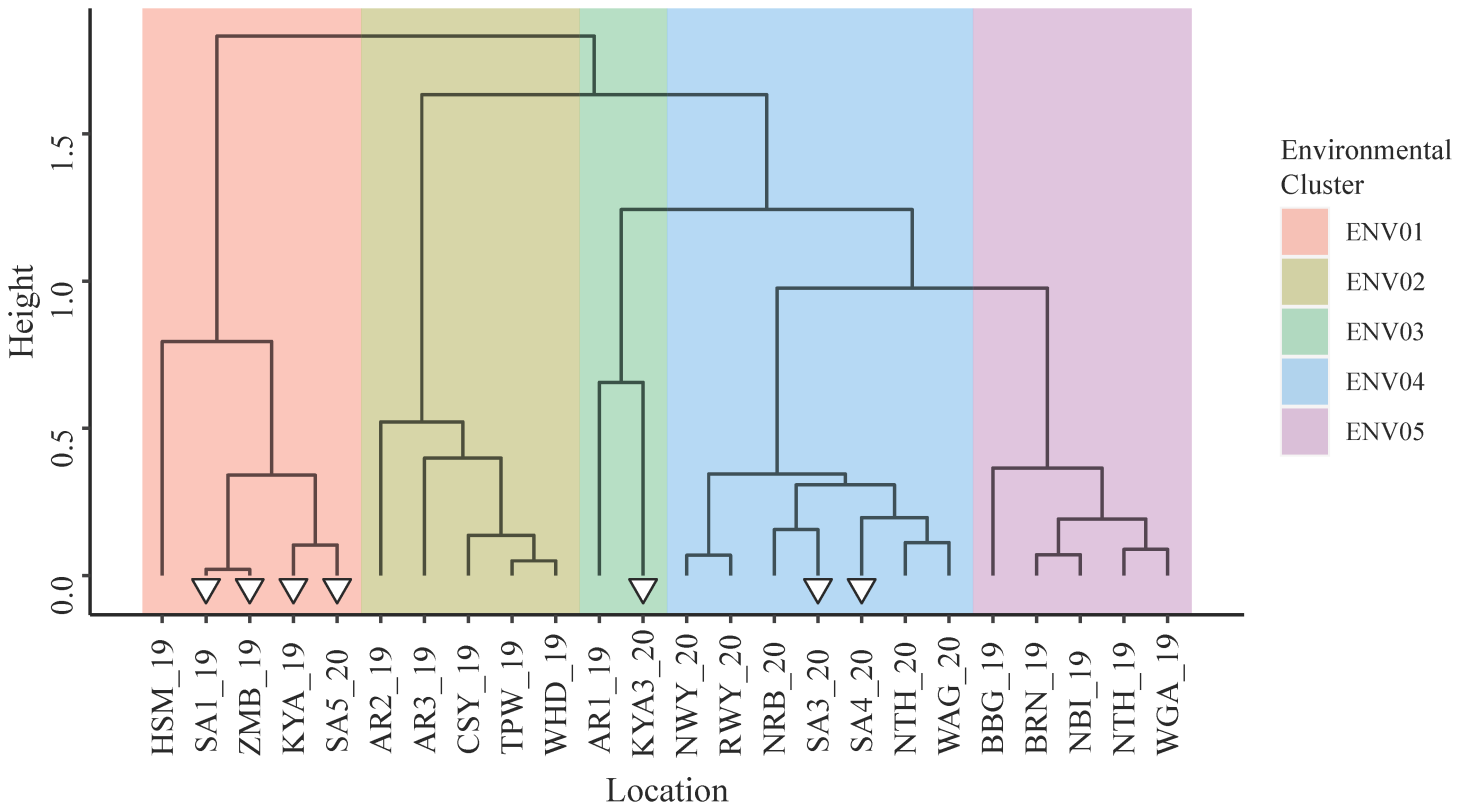
Environmental clusters revealed by hierarchical clustering of the additive genetic correlation matrix formed from the additive genetic variances and covariances between trial sites. White triangles identify trial sites within Sub-Saharan Africa.

**Supplementary Table 8** provides an overview of the trial statistics associated with each trial site. Additive genetic variance was the main contributor to phenotypic variance in 20 of the 24 sites. In total, 68.8% of the additive genetic variance was explained by the first two factor loadings of the FA4 model (**Supplementary Figure 1**). Hierarchical clustering of the additive genetic correlation matrix revealed five distinct environmental clusters (**Figure 2**); large genotype by environment interactions (GxE) were observed (**Supplementary Table 7**). With the meteorological data available, no clear differences were observed between the declared environmental clusters (**Supplementary Table 9**).

Southern and Eastern African trial sites were found within clusters ENV01, ENV03 and ENV04. There was a notable absence of European trial sites within these clusters; in general, negative correlations were observed between the additive genetic effects of parents in the European sites and those in Africa (**Supplementary Table 7**). In the remainder of this paper, only parental and marker effects for clusters ENV01 and ENV04 are discussed for the sake of brevity; ENV03 contained the African site KYA3_20 but is excluded from discussion as the additive genetic variance observed in KYA3_20 was less than 20%.

ENV04 was characterized by higher grain yield potential than ENV01; the latter being characterized by a relatively shorter time from sowing to harvest (**Supplementary Table 8**). In both environments, test hybrids with yield exceeding the mean performance of the inbred checks were observed (**Supplementary Figure 2**); hybrids outperforming the best inbred check were observed at the SA3_20 and ZMB_19 trial sites.

### 3.2 Genetic relationships between parents

**Figure 3:**
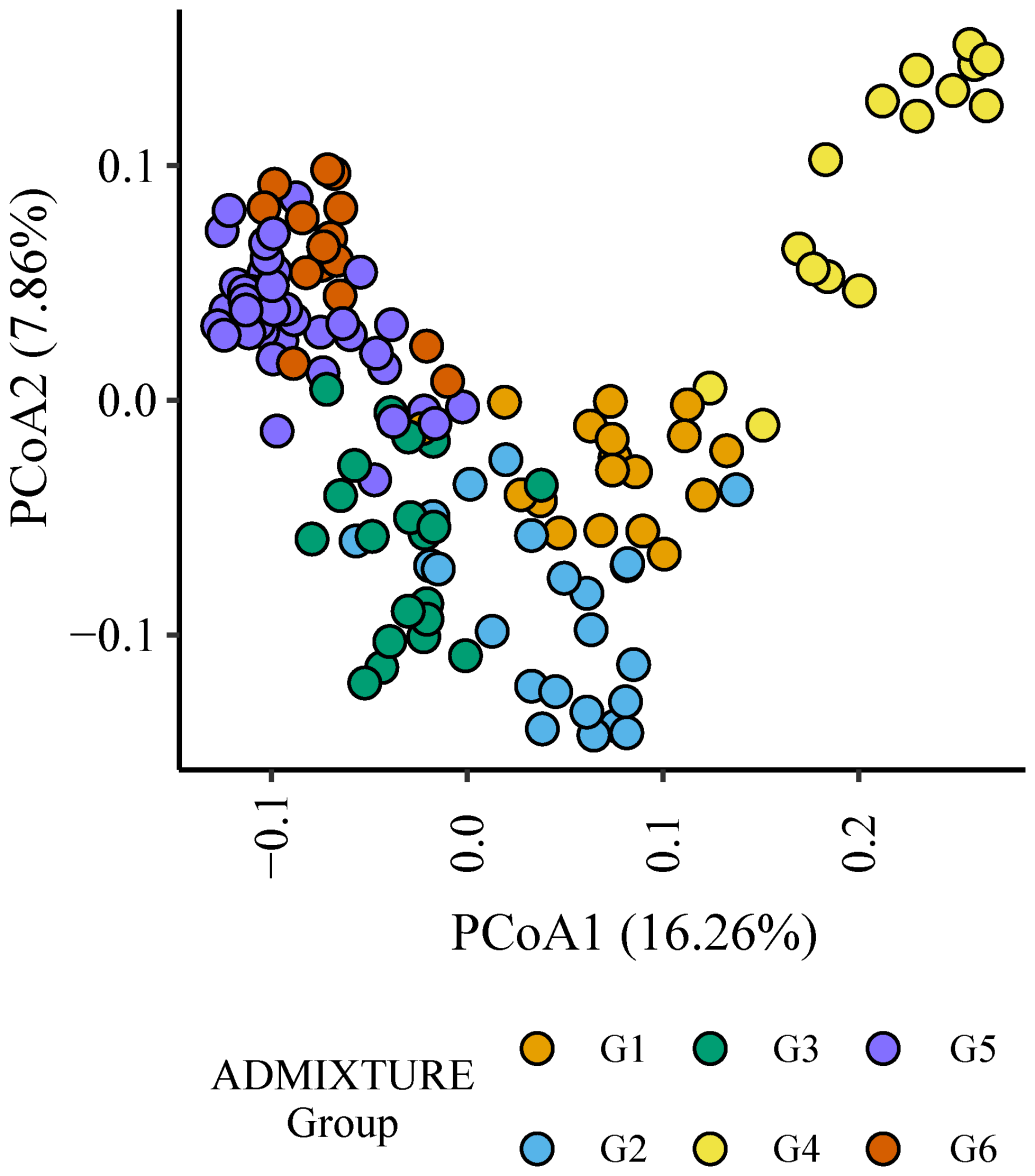
Plot of principal coordinates one (1) and two (2) derived from the Roger’s genetic distance between parents. The colour of each point indicates the genetic subgroups identified by ADMIXTURE analysis.

Six (G1 to G6) distinct genetic subgroups were identified among the parents in this study (**Figure 3**, **Table 1**). European germplasm (G4) formed a distinct cluster that was confirmed by principal coordinate analysis of Roger’s genetic distance values (**Figure 3**). A greater level of admixture was observed among remaining groups (**Figure 3**) indicative of germplasm exchange between agroclimatic zones.

**Table 1:** Genetic subgroups identified among parental components by ADMIXTURE analysis.

Genetic subgroup	N	Description
G1	18	Female components with Chinese ancestry and modern Chinese germplasm.
G2	23	Argentinian elites, North American elites bred for the hard red spring region of the United States of America, female components derived from these North American elites and South African facultative elites.
G3	20	South African elites bred for irrigated and rainfed conditions and Argentinian elites.
G4	15	German and UK bred elites and female components derived from this germplasm.
G5	42	CIMMYT elites bred for high rainfall environments and Argentinian elites.
G6	16	CIMMYT elites bred for high rainfall environments and a single Australian bred female component.

The number (N) of individuals within each subgroup and a brief description of the germplasm within each is provided.

### 3.3 Additive effect of parents in environmental clusters ENV01 and ENV04 in relation to genetic subgroup

**Figure 4:**
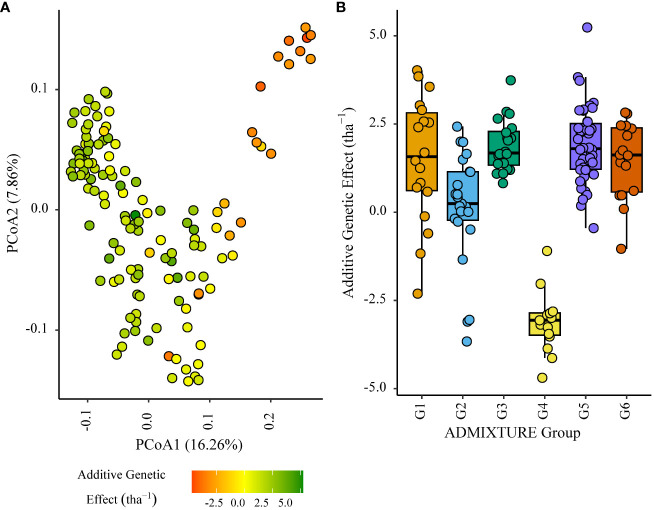
Additive genetic effect (BLUP) for grain yield (tha^-1^) associated with parents in trial site SA1_19 (ENV01); **(A)** overlaid on plot of principal coordinate one (1) and two (2) derived from the Roger’s genetic distance between parents and **(B)** displayed as boxplots with genetic subgroup, from ADMIXTURE analysis, as the grouping factor.

**Figure 5:**
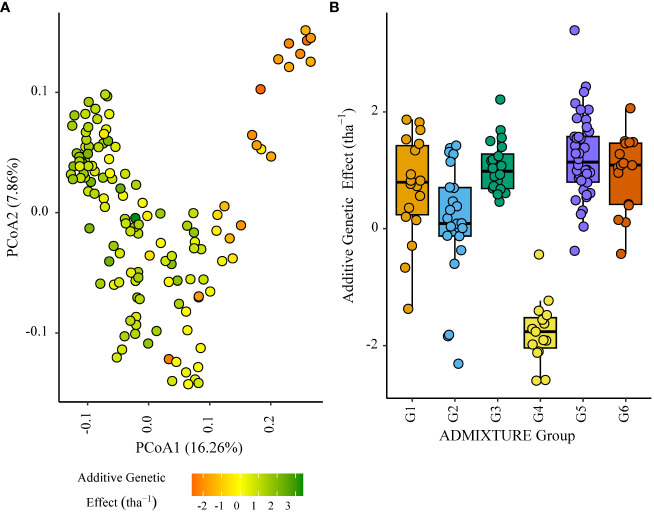
Additive genetic effect (BLUP) for grain yield (tha(tha^-1^) associated with parents in trial site ZMB_19 (ENV01); **(A)** overlaid on plot of principal coordinate one (1) and two (2) derived from the Roger’s genetic distance between parents and **(B)** displayed as boxplots with genetic subgroup, from ADMIXTURE analysis, as the grouping factor.

**Figure 6:**
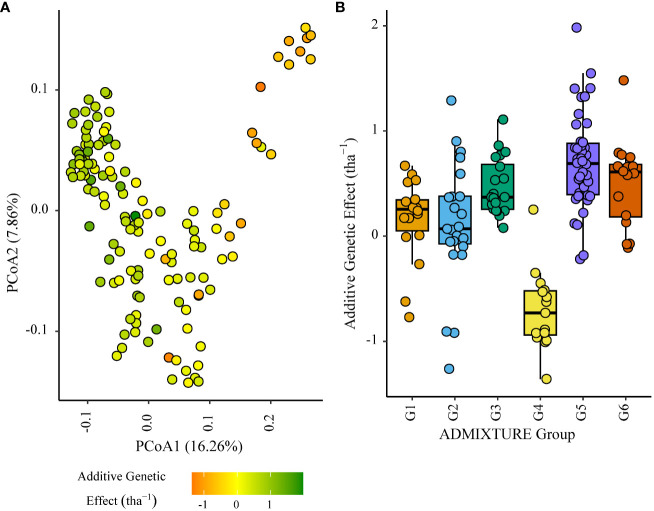
Additive genetic effect (BLUP) for grain yield (tha(tha^-1^) associated with parents in trial site KYA_19 (ENV01); **(A)** overlaid on plot of principal coordinate one (1) and two (2) derived from the Roger’s genetic distance between parents and **(B)** displayed as boxplots with genetic subgroup, from ADMIXTURE analysis, as the grouping factor.

**Figure 7:**
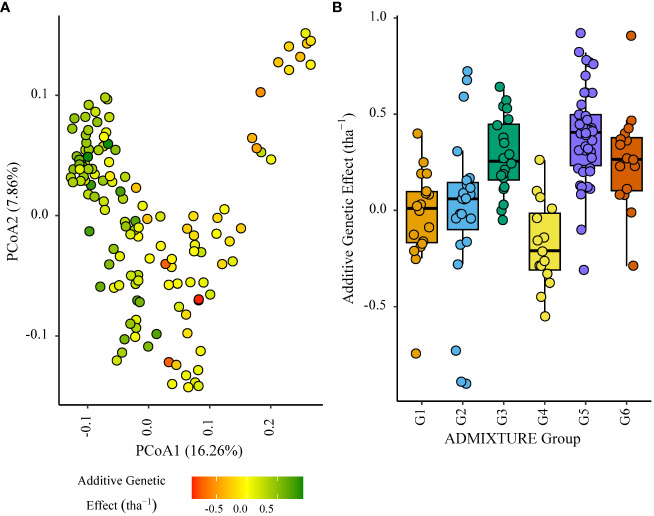
Additive genetic effect (BLUP) for grain yield (tha^-1^) associated with parents in trial site SA5_20 (ENV01); **(A)** overlaid on plot of principal coordinate one (1) and two (2) derived from the Roger’s genetic distance between parents and **(B)** displayed as boxplots with genetic subgroup, from ADMIXTURE analysis, as the grouping factor.

Within the African trial sites belonging to ENV01, parents with a positive additive effect for grain yield were observed in each genetic subgroup; such individuals were less common in the European subgroup G4 (**Figures 4**–**7**). Parents belonging to G5 showed additive effects surpassing those observed for G3 individuals, the latter having been bred for the environments in this cluster.

**Figure 8:**
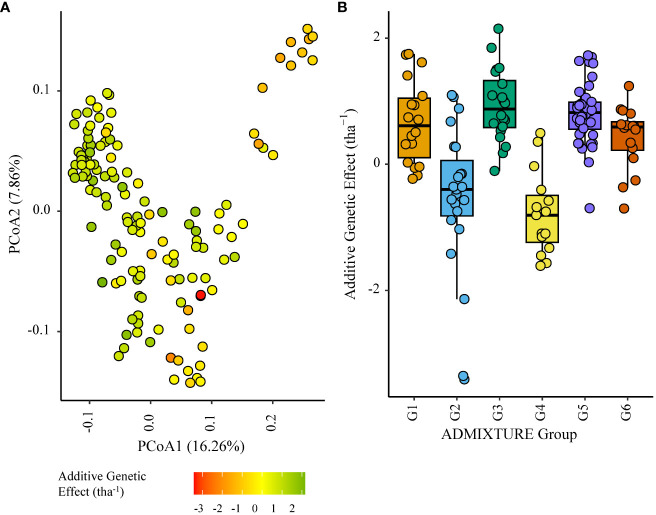
Additive genetic effect (BLUP) for grain yield (tha^-1^) associated with parents in trial site SA3_20 (ENV04); **(A)** overlaid on plot of principal coordinate one (1) and two (2) derived from the Roger’s genetic distance between parents and **(B)** displayed as boxplots with genetic subgroup, from ADMIXTURE analysis, as the grouping factor.

**Figure 9:**
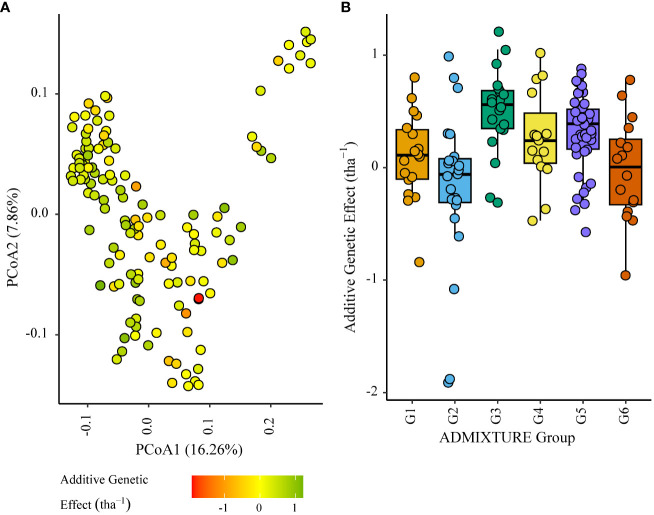
Additive genetic effect (BLUP) for grain yield (tha^-1^) associated with parents in trial site SA4_20 (ENV04); **(A)** overlaid on plot of principal coordinate one (1) and two (2) derived from the Roger’s genetic distance between parents and **(B)** displayed as boxplots with genetic subgroup, from ADMIXTURE analysis, as the grouping factor.

G4 individuals negatively impacted grain yield to a lesser extent in ENV04, with some showing relatively high effects in SA4_20. The highest additive effects were observed in the South African elite subgroup G3 (**Figures 8**, **9**).

**Figure 10:**
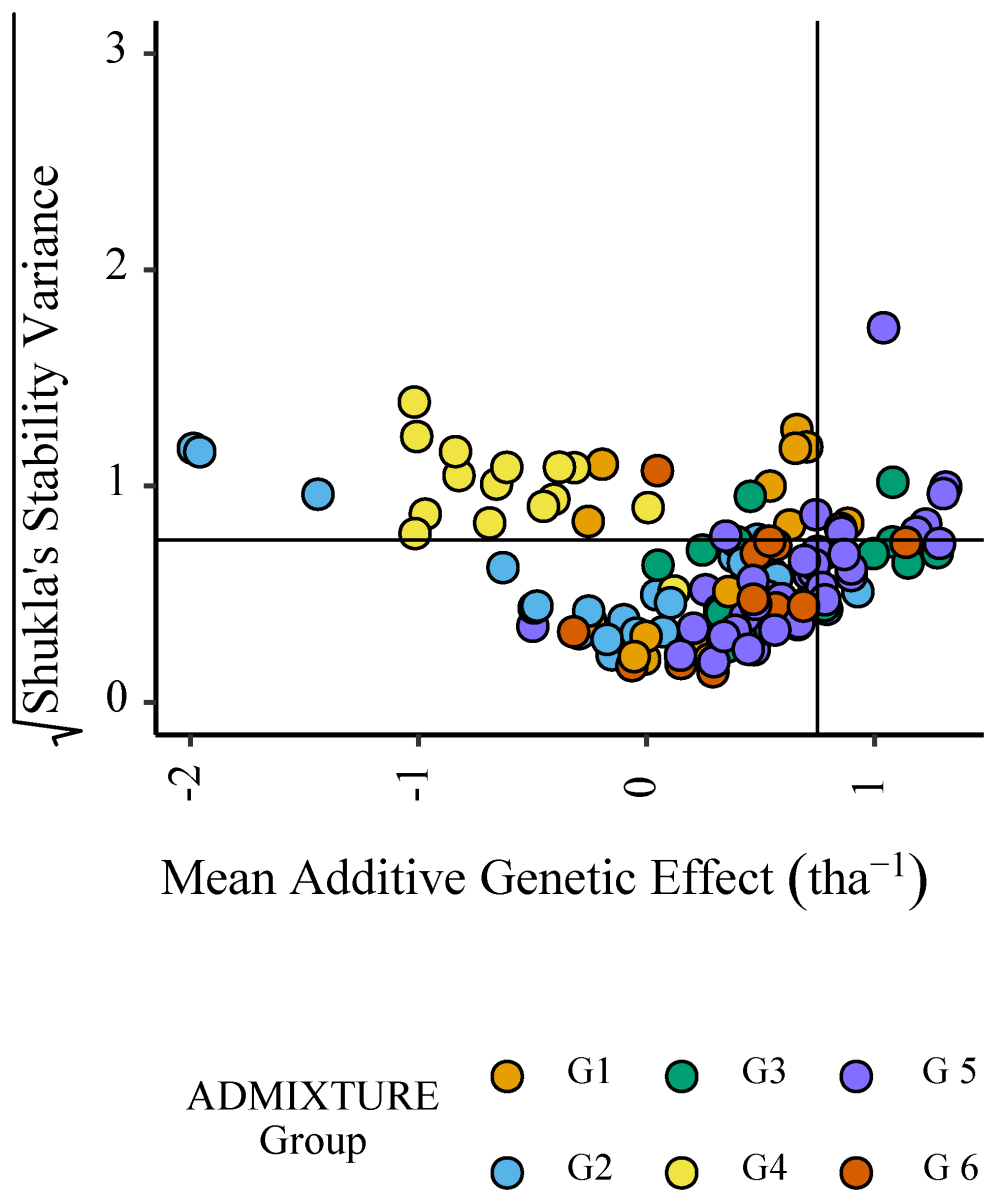
Mean additive genetic effect (BLUP) for grain yield (tha^-1^) of parents in ENV01 and ENV04 plotted against the square root of Shukla’s stability variance; both parameters calculated using all trial sites in ENV01 and ENV04. Colour of points indicate the genetic subgroup of individuals.

The stability of parental effects across trial sites in ENV01 and ENV04 was explored by plotting the mean additive effect of each parent against the square root of Shukla’s stability variance. Dividing the plot into quadrants identified parents with a stable positive contribution to grain yield (upper right quadrant); these individuals belonged to genetic subgroups G1, G3, G5 and G6 (**Figure 10**).

### 3.4 Genomic distribution of additive effects for grain yield (ENV01 and ENV04)

**Figure 11:**
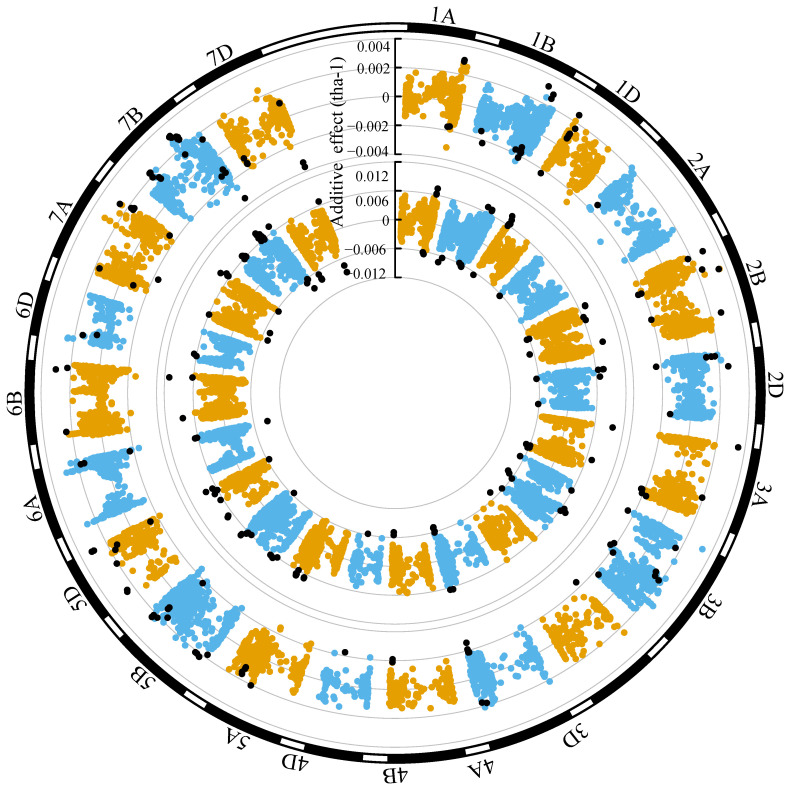
Circular manhattan plot of the additive effects for grain yield (tha^-1^) associated with SNP markers for SA1_19 (inner circle; ENV01) and KYA_19 (outer circle; ENV01). The top ten percent of SNPs showing the largest effects (absolute) in SA1_19 is shown using black dots in both circles, to visualize the extent to which effects are shared between the two trial sites.

**Figure 12:**
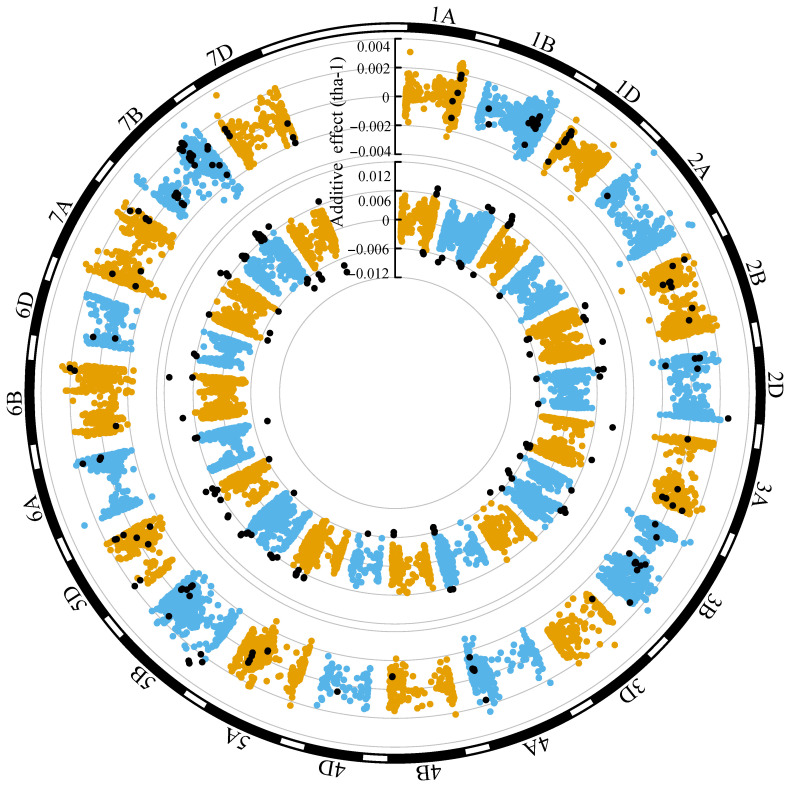
Circular manhattan plot of the additive effects for grain yield (tha^-1^) associated with SNP markers for SA1_19 (inner circle; ENV01) and SA4_20 (outer circle; ENV04). The top ten percent of SNPs showing the largest effects (absolute) in SA1_19 is shown using black dots in both circles, to visualize the extent to which effects are shared between the two trial sites.

Grain yield marker effects in both ENV01 and ENV04 were distributed throughout the genome (**Figures 11**, **12**). We observed that marker effects were relatively consistent between trial sites within the same environmental cluster, e.g. SA1_19 (ENV01) and KYA_19 (ENV01) (**Figure 11**). Comparison between trial sites belonging to different clusters revealed differences in marker effects with respect to their sign (positive vs negative) and magnitude of their effect, e.g. SA1_19 (ENV01) and SA4_20 (ENV04) (**Figure 12**).

In trial sites belonging to ENV01 (KYA_19, SA1_19, SA5_20 and ZMB_19), KASPs targeting *PPD-D1* and *VRN-A1* were in the upper distribution of marker effects; in ENV04 (SA3_20 and SA4_20), KASPs targeting *VRN-B1* and *SM1* were in the upper distribution (**Supplementary Figure 3**). A strong effect was associated with the KASP targeting *YR5* in KYA_19, SA4_20 and SA5_20 (**Supplementary Figure 3**).

### 3.5 Variation in usefulness criterion associated with genetic subgroups of parents (ENV01 and ENV04)

**Figure 13:**
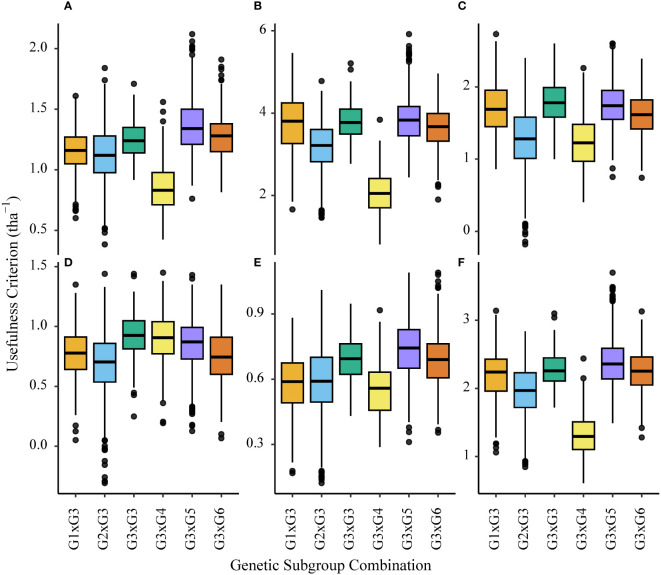
Boxplots showing variance in the usefulness criterion of simulated interpopulation (G3 x any other group) and intrapopulation (G3 x G3) crosses made with African germplasm (G3). Results using marker effects specific to each African trial site are shown; **(A)** KYA_19 (ENV01), **(B)** SA1_19 (ENV01), **(C)** SA3_20 (ENV04), **(D)** SA4_20 (ENV04), **(E)** SA5_20 (ENV01) and **(F)** ZMB_19 (ENV01).

For ENV01 trial sites (KYA_19, SA1_19, SA5_20 and ZMB_19) the usefulness criterion was highest for interpopulation crosses where genetic subgroup G3 was the anchor; specifically, simulated populations between G3 and G5/G6 parents outperformed those between G3 and G3 parents (**Figure 13**). This trend was not observed for the two African trial sites in ENV04, SA3_20 and SA4_20 (**Figure 13**).

## 4 Discussion

We assessed the performance of a diverse panel of inbred spring wheat lines for their additive effect on grain yield in Southern and Eastern Africa by multisite testing of their F1 test hybrids. Considerable GxE interactions were observed within our network of trial sites. Testing in South America and Europe appears to be unsuitable for selecting hybrids suited to ENV01 or ENV04; Australian trial sites appear to be of greater utility, especially for ENV04. Collaboration between entities in Australia and those in Sub-Saharan African, either in the form of public-private partnerships or commercial partnerships could be a viable approach to leverage Australian infrastructure and germplasm in the re-establishment of hybrid wheat breeding in Sub-Saharan Africa.

ENV01 trial sites were characterized by a shorter period from sowing to harvest; temporal variation in the environmental variables accessed did not form discreet patterns that matched the declared environmental clusters. It is possible that within a cluster, different environmental variables have influenced the duration of the growing season; additionally, the interplay between these variables and additional factors such as soil characteristics may explain our observations. Within ENV01 and ENV04 we observed distinct differences in the ability of parents to contribute positively to F1 grain yield related to the genetic subgroup in which they fall. The implications of this for exploiting regional heterosis are discussed.

There are two prevailing explanations for heterosis in literature: the overdominance theory and the directional dominance theory. The overdominance theory implies an inherent advantage of the heterozygous state over homozygosity. While there are examples of overdominance at single loci in crop species (Mackay et al., 2021), investigations into hybrids formed between heterotic pools have found overdominance to be a rare phenomenon (Cowling et al., 2020; Mackay et al., 2021). In some instances, what was described as single locus overdominance was later found to be caused by dispersed dominant loci in high linkage disequilibrium with each other, a phenomenon described as pseudo-overdominance (Jones, 1917).

The directional dominance theory is an extension of an additive model of gene action which incorporates dominance. With this theory, it is the presence of an excess of loci showing partial dominance in the same direction that enables a hybrid to outperform its parents. Heterotic pool formation would therefore be an exercise in maximizing differences in the allelic frequency of loci, fitting this criterion, between two or more germplasm groups. While this model is simplistic and does not account for epistatic gene interactions, it often fits observed patterns of genetic segregation, genetic gain and mid parent heterosis (Cowling et al., 2020; Mackay et al., 2021). Nevertheless, Jiang et al. (2017) reported that epistatic interactions were a major contributor to heterosis in wheat. We do not find this to be the case in the present study, with additive genetic variance prevailing in most trial sites; we attribute this to the larger genetic diversity in our parental panel and the expectation that additive effects will outweigh non-additive effects with genetically divergent groups (Labroo et al., 2021).

Bernardo (2002) described the four criteria that are most useful in identifying heterotic groups and patterns as; 1) high mean performance and large genetic variance in the cross between heterotic groups (i.e. high usefulness), 2) high per se performance and good adaptation of the heterotic groups, 3) ability to maintain and propagate inbred parental components and 4) availability of an efficient sterility system. In wheat, criteria three (3) and four (4) should not be viewed as roadblocks as the species reliably self-pollinates and stable sterility systems are available (Whitford et al., 2013; Sun et al., 2019). The work of organizations such as CIMMYT demonstrates that elite cultivars can be bred for broad adaptation without compromising yield potential (Braun et al., 1997) and this addresses criterion two (2). Our study provides evidence that criterion one (1) can also be met with elite spring wheat germplasm that is available across the globe.

European germplasm (G4) would generate hybrids with a large genetic variance if mated with germplasm adapted to Africa (G3). This fulfills one part of Bernardo’s first criterion; however, this approach will fail to produce hybrids with a high mean performance due to the negative additive effects associated with G4 in African trial sites. Genetic isolation is expected to lead to a divergence in allele frequencies; if two populations are grown in contrasting environments, they may be selected for different trait profiles and the resulting pattern of allelic divergence can compromise the fitness of inter-population single crosses in both environments (Mackay et al., 2021). The extent of negative correlations between the additive effects of European trial sites and African trial sites implies that distinct trait profiles are needed for the two regions.

Using African germplasm (G3) as an anchor, we anticipate that using parents from either G5 and/or G6 to develop a complementary pool would produce hybrids with a relatively high genetic variance and a high mean performance. In ENV01, simulations of UC in interpopulation and intrapopulation crosses involving G3 supported this; indicating that some of the genetic factors underlying the positive additive effect of parents from G3 and G5/6 are unique to their respective subgroups. The divergence observed between these genetic subgroups has occurred in a manner that is useful for hybrid breeding in the region. The wide distribution of positive additive effects throughout the genome implies that the development of inter-population hybrids would be more efficient than attempting to combine the beneficial alleles of these subgroups via the development of recombinant inbred lines. With the germplasm in our study, the value of inter-population hybrids was less evident in ENV04; reciprocal recurrent selection (Labroo et al., 2021) within G5 should be sufficient to form a heterotic pool that better complements one based on G3 germplasm in ENV04; beneficial genetics from high performing individuals of G1, G2 or G6 could also be leveraged to accelerate this process if necessary.

It is interesting that CIMMYT high rainfall germplasm (G5 and G6) showed stable positive effects when used as hybrid parents; it was established that this material can show high yield per se and high yield stability simultaneously (Braun et al., 1997). The broad-adaptation of CIMMYT germplasm seemingly translates into stable parental effects; this is of immense value to hybrid breeding for ENV01 and ENV04 as a genetically distinct germplasm pool that shows similar levels of adaption to that of local germplasm is already available. The simulations performed by Cowling et al. (2020) showed that over 30 cycles of recurrent selection, random splitting of founders performed equally to separation into heterotic pools based on GCA and genetic distance. In the short term (5 cycles and assuming a complex genetic architecture), there was a yield advantage when the latter method was employed; this may be required in commercial breeding where a return on investment in the shortest time is desirable.

We should be careful not to dismiss the possibility that G4 can contribute positively in this region; these materials may harbor beneficial alleles that are not present in either the CIMMYT or African germplasm. Intuitively, having the wrong profile of adaptation alleles prohibited G4 germplasm from showing a net positive effect in the region. The shorter growing seasons observed in ENV01and the pronounced effect of the *PPD-D1* locus in this environmental cluster supports this; the longer growing seasons (more similar to those in Europe) within ENV04 provide an explanation for G4 germplasm having less of a negative impact in this cluster. The variation in additive effects observed within G4 suggests that despite lacking the correct adaptation profile, some of the germplasm may have positive effects that are being masked by phenology and/or agronomic traits. Pre-breeding of inbred crop species demonstrates that adaptation can be decoupled from yield potential (Bernardo, 2002) and the work of Koekemoer et al. (2011) showed that differences in vernalization and photoperiod requirement between pools can be overcome in hybrid breeding. G4 lines can be used as a genetic resource to increase the frequency of beneficial alleles in either of our hypothetical pools prior to the application of reciprocal recurrent selection (Cowling et al., 2020).

There is no strong evidence for commercial heterosis in our dataset and thus the utility of hybrid spring wheat breeding to increase yield can be questioned. We argue that the results should not be interpreted in this way; rather, the fact that test hybrids produced with little prior information, using diverse parents, can be competitive with established inbred material, supports the potential of hybrid wheat for increasing yield in Southern and Eastern Africa. Undoubtedly, with sustained breeding and the establishment of heterotic pools we will see considerable improvement in the performance of F1 hybrids over time in this region.

## Data availability statement

The raw data supporting the conclusions of this article will be made available by the authors, without undue reservation.

## Author contributions

CJ-B: Data curation, Investigation, Methodology, Project administration, Visualization, Writing – original draft, Writing – review & editing. RT: Conceptualization, Funding acquisition, Methodology, Resources, Supervision, Writing – review & editing. IR: Data curation, Investigation, Methodology, Writing – review & editing. SdG: Investigation, Methodology, Writing – review & editing. LS: Investigation, Methodology, Writing – review & editing. FK: Funding acquisition, Investigation, Methodology, Resources, Supervision, Writing – review & editing. SD: Data curation, Formal analysis, Writing – review & editing. JL: Funding acquisition, Methodology, Resources, Supervision, Writing – review & editing.
